# Cell-Type-Specific Changes in Intrinsic Excitability in the Subiculum following Learning and Exposure to Novel Environmental Contexts

**DOI:** 10.1523/ENEURO.0484-18.2018

**Published:** 2019-01-04

**Authors:** Amy R. Dunn, Sarah M. Neuner, Shengyuan Ding, Kevin A. Hope, Kristen M.S. O’Connell, Catherine C. Kaczorowski

**Affiliations:** 1 The Jackson Laboratory, Bar Harbor, ME 04609; 2 University of Tennessee Health Sciences Center, Memphis, TN 38136

**Keywords:** burst spiking, contextual fear conditioning, intrinsic excitability, plasticity, regular spiking, subiculum

## Abstract

The subiculum is the main target of the hippocampal region CA1 and is the principle output region of the hippocampus. The subiculum is critical to learning and memory, although it has been relatively understudied. There are two functional types of principle neurons within the subiculum: regular spiking (RS) and burst spiking (BS) neurons. To determine whether these cell types are differentially modified by learning-related experience, we performed whole-cell patch clamp recordings from male mouse brain slices following contextual fear conditioning (FC) and memory retrieval relative to a number of control behavioral paradigms. RS cells, but not BS cells, displayed a greater degree of experience-related plasticity in intrinsic excitability measures [afterhyperpolarization (AHP), input resistance (R_input_), current required to elicit a spike], with fear conditioned animals having generally more excitable RS cells compared to naïve controls. Furthermore, we found that the relative proportion of RS to BS neurons is modified by the type of exposure, with the lowest proportion of BS subicular cells occurring in animals that underwent contextual FC followed by a retrieval test. These studies indicate that pyramidal neurons in the subiculum undergo experience- and learning-related plasticity in intrinsic properties in a cell-type-specific manner. As BS and RS cells are thought to convey distinct types of information, this plasticity may be particularly important in encoding, consolidating, and recalling spatial information by modulating information flow from the hippocampus to cortical regions.

## Significance Statement

The subiculum is the main output region of the hippocampus and contains two main neuron types: regular spiking (RS) and burst spiking (BS). Here, we show that learning and novel context exposure induces plasticity in intrinsic excitability in RS cells of the subiculum and that this plasticity predicts degree of learning of contextual fear conditioning (FC). In contrast, we observed no significant learning-induced plasticity in intrinsic properties in subicular BS cells; however, we did observe an apparent conversion of BS to RS as evidenced by a greater proportion of RS neurons within the subiculum following learning. This suggests that the ratio of RS to BS neurons within the subiculum, together with enhanced excitability of RS neurons, is important in learning and memory.

## Introduction

The subiculum plays a pivotal role in conveying information from the hippocampus to cortical and subcortical regions, receiving and integrating information directly from hippocampal region CA1 (Naber and Witter, 1998). The subiculum mediates various aspects of behavior including spatial and declarative memory, contextual fear memory, memory retrieval, discrimination of complex scenes, temporal control of behavior, and motivated behavior (Gabrieli et al., 1997; Maren, 1999; O'Mara et al., 2009; Hodgetts et al., 2017; Roy et al., 2017; Cembrowski et al., 2018). In humans, it is involved in both normal cognitive function and in diseases characterized by impaired cognition including schizophrenia and Alzheimer’s disease (Carlesimo et al., 2015; Haukvik et al., 2015; Lindberg et al., 2017; Zammit et al., 2017). The subiculum is organized in both proximal-distal and dorsal-ventral directions, with heterogenous inputs and outputs, cell types, cytoarchitecture, contributions to behavior, and gene and protein expression along each axis (O'Mara, 2005; O'Mara et al., 2009; Kim and Spruston, 2012; Honda and Ishizuka, 2015; Ishihara and Fukuda, 2016; Tang et al., 2016; Roy et al., 2017; Cembrowski et al., 2018). This complex organization likely allows for various parallel information processing pathways flowing from the hippocampus.

The subiculum has two pyramidal neuron types, burst spiking (BS) and regular spiking (RS) cells. Previous studies indicate that BS cells make up about one-half of pyramidal neurons in the subiculum. BS cells are not evenly distributed throughout the subiculum (Staff et al., 2000), with the density of BS cells gradually increasing along the proximal-distal axis (Jarsky et al., 2008). RS and BS cells show differences in synaptic plasticity (Wozny et al., 2008; Behr et al., 2009; Shor et al., 2009; Graves et al., 2016), as well as differences in intrinsic excitability (Staff et al., 2000; Behr et al., 2009). The differences in plasticity, pathways, and excitability of these cell types suggest meaningful differences in how and what information is encoded by BS and RS cells, allowing the subiculum to regulate learning- and memory-related information conveyance from the hippocampus to the rest of the brain.

Plasticity in intrinsic excitability is an important mechanism of learning and memory (Titley et al., 2017) and arises from changes to ion channel function and/or distribution on the surface of neuronal membranes. Changes in intrinsic excitability may occur independently of synaptic plasticity, but can also modulate synaptic plasticity (Sehgal et al., 2013). The relationship between learning and intrinsic properties of neurons has been extensively studied throughout the brain, including CA1, the amygdala (Sehgal et al., 2014), and the cortex (Song et al., 2015; Soler-Cedeño et al., 2016). Learning and environmental enrichment generally result in greater neuronal excitability across species (Kumar and Foster, 2007; Oh et al., 2009; Matthews et al., 2009; Song et al., 2012), and enhanced intrinsic excitability as measured by a reduced afterhyperpolarization (AHP) is associated with better learning and memory (Kaczorowski and Disterhoft, 2009). However, it is unknown how learning alters intrinsic excitability of subicular neurons. Given the diversity in subicular pyramidal neurons, and their importance in integrating and transmitting information from the hippocampus, determining how learning remodels the subiculum is critical to a more complete understanding of how learning and memory is encoded in the brain.

Here, we examined how hippocampal-dependent contextual fear conditioning (FC; Phillips and LeDoux, 1992) modifies intrinsic excitability properties of subicular RS and BS cells. We observed enhanced excitability across various measures following learning compared to naïve animals, and these learning-related changes were specific to RS cells. Greater excitability in RS cells was associated with better performance on long-term contextual memory. Finally, we found that learning results in an increase in the proportion of RS cells, suggesting interconversion between BS and RS cells *in vivo* following context encoding and recall. Our study supports previous research demonstrating differential plasticity in RS and BS, and confirms that the subiculum undergoes cell-type-specific plasticity in intrinsic properties following novel context encoding and fear learning. Overall, we found that experience-dependent remodeling of RS cells may be important in generating new learning and contextual memory related information.

## Materials and Methods

### Animals

Adult male (seven to eight weeks) C57BL/6J were obtained from the live repository at The Jackson Laboratory (JAX; RRID:IMSR_JAX:000664) and housed on a 12/12 h light/dark cycle with *ad libitum* access to food and water. All experiments occurred at JAX or the University of Tennessee Health Science Center (UTHSC) and were conducted in accordance with the JAX and UTHSC Animal Care and Use Committee and the National Institutes of Health Guide for the Care and Use of Laboratory Animals.

### Behavioral paradigms

**Figure 1.**
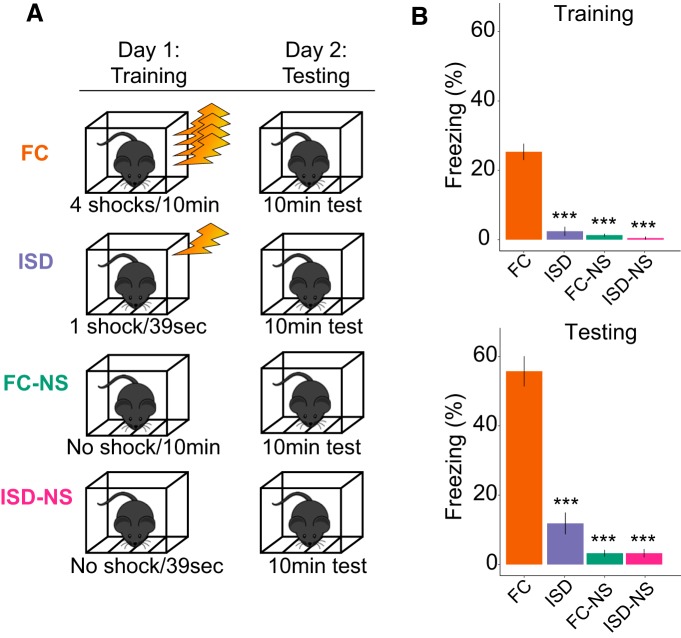
Contextual FC results in greater fear memory compared to control groups. ***A***, Animals were randomly assigned to one of several behavioral training paradigms including: FC, ISD, FC-NS, and ISD-NS. ***B***, Degree of freezing during training and during the testing period the following day were assessed. Animals in the FC condition froze significantly more than any other group on both training and testing days, as expected; ***p < 0.001.

Animals were randomly assigned to behavioral paradigms (Fig. 1*A*, schematic), and underwent the following behavioral testing.

#### Contextual FC

Animals were habituated to the behavioral testing facility for at least 3 d before training. Specifically, animals were transported in their home cages to a holding room separated from the testing room for 1 h/d before testing. Because mice were group-housed within their home cages and only one animal per day was tested, cagemates awaiting testing were necessarily habituated for additional days (up to 10 additional days). Mice were trained on a standard contextual FC paradigm as described previously (Neuner et al., 2015). Briefly, animals were placed in the conditioning chambers. Following a 150-s baseline period, animals received four mild foot shocks (1 s, 0.9 mA) separated by 150 ± 25 s over 10 min. The 20 s following each shock was designated as the postshock period, and freezing during each postshock period was quantified. Twenty-four hours later, animals were returned to the chambers for 10 min. Percentage time spent freezing during this time was measured using FreezeFrame software (ActiMetrics; RRID:SCR_014429) and used as an index of long-term contextual memory, consolidation and retrieval. Immediately after testing, animals were anaesthetized using isoflurane and hippocampal slices harvested for electrophysiological analysis.

#### Immediate shock deficit (ISD)

Animals were habituated to the behavioral testing facility for at least 3 d before training. Animals were placed in the conditioning chamber, immediately received a mild foot shock (4 s, 0.9 mA), and were rapidly removed from the chamber, for a total of 39 s spent in the conditioning chamber. Twenty-four hours later, animals returned to the chambers for 10 min. Immediately following testing, animals were anaesthetized using isoflurane and hippocampal slices harvested for electrophysiological analysis. This provided a control for exposure to the stress of receiving foot shocks for 4-s total in experimental groups (Neuner et al., 2015).

#### FC–no shock (FC-NS) control

Animals were habituated to the behavioral testing facility for at least 3 d before training. Animals were allowed to explore the conditioning chamber for 10 min with no foot shock. Twenty-four hours after “training,” animals were again allowed to explore the conditioning chambers for 10 min. Immediately after testing, animals were anaesthetized using isoflurane and hippocampal slices harvested for electrophysiological analysis. This provided a no-shock control to the FC group.

#### ISD–no shock (ISD-NS) control

Animals were habituated to the behavioral testing facility for at least 3 d before training. Animals were placed in the FC chambers for 39 s with no shock, and returned to the chambers 24 h later for 10 min. This provided a no-shock control for the ISD group. Immediately after testing, animals were anaesthetized using isoflurane and hippocampal slices harvested for electrophysiological analysis.

#### Naïve

Animals remained in the housing facility for the duration of the experiment and were removed only for electrophysiological analysis.

### Electrophysiology

Hippocampal slices were prepared and whole-cell patch clamp recordings were performed from subicular neurons as described previously (Graves et al., 2016). Mice were deeply anaesthetized with isoflurane and decapitated. Brains were removed and placed in ice-cold artificial CSF (aCSF; 125 mM NaCl, 25 mM glucose, 25 mM NaHCO_3_, 2.5 mM KCl, 1.25 mM NaH_2_PO_4_, 2 mM CaCl_2_, 1 mM MgCl_2_, saturated with 95% O_2_/5% CO_2_); 300 μM transverse sections were made with a vibratome (Leica, VT1000S). Slices were incubated in aCSF bubbled with 95% O_2_/5% CO_2_ at room temperature for at least 1 h before use. Slices were transferred to a recording chamber and continuously perfused with oxygenated aCSF at ∼30°C. Synaptic blockers including 1–2 mM kynurenic acid (NMDA receptor antagonist, to block excitatory input) and 2 μM SR95531 (GABA-A receptor antagonist, to block inhibitory input) were included in aCSF in initial studies but discontinued when no statistically significant differences in the intrinsic properties was observed compared to aCSF alone, consistent with prior studies of burst plasticity using learning-relevant electrical stimulation (Moore et al., 2009; Graves et al., 2012). Whole-cell recordings were made in the subiculum from thin-walled capillary glass pipettes (Sutter Instrument Co, P-97 and P-2000) filled with potassium gluconate based internal solution (115 mM K-gluconate, 20 mM KCl, 10 mM Na-phosphocreatine, 10 mM HEPES, 2 mM MgATP, and 0.3 mM NaGTP, pH adjusted to 7.3). Pyramidal neurons in the subiculum were visually identified with a Q-Imaging digital camera and DIC-IR microscopy. Data were acquired using a MultiClamp 700B amplifier, digitized and interfaced to PC running pClamp10.4 with Axon Digidata 1550B analog to digital converter. Current clamp experiments were conducted to determine whether the excitability of pyramidal neurons in the subiculum are modulated by FC training. For assessment of intrinsic excitability (AHP, I_thresh_, etc.), neurons were held at –67 mV with current injection. Series resistance and capacitance were monitored and compensated for throughout the recording. Membrane potentials were not corrected for the liquid junction potential, which was estimated to be −8 mV (Kaczorowski et al., 2007). Up to four subiculum cells per slice were recorded. Because there is not a well-defined boundary between distal and proximal subiculum, and various neuronal properties vary across a gradient from the proximal subiculum (i.e., adjacent to CA1) and distal subiculum (i.e., adjacent to the presubiculum), cells were chosen at random equally across the proximal-distal length of the subiculum. This random sampling procedure within the subiculum of naïve rodents has consistently identified ∼50% BS cells by our lab and others (Staff et al., 2000; Jarsky et al., 2008; Graves et al., 2016).

#### Neuronal classification

The subiculum contains two functionally distinct principle cell types. to test the hypothesis that neurons in the subiculum undergo learning-related changes in excitability, and determine whether these changes depend on cell type, neurons were classified as RS or BS based on their response to a 2-ms current pulse at threshold. RS neurons fired a single action potential in response to injected current at threshold, whereas BS neurons fired two or more action potentials.

#### Assessing neuronal excitability

Resting membrane potential (RMP) was recorded within five minutes of patching each cell. Input resistance (R_input_) was calculated using Ohm’s law and determined by fitting the slope of the IV plot from the membrane response to 1-s current injections in 10-pA steps (from –50 to +50 pA) at steady-state (i.e., last 100 ms of trace). The current threshold (I_thresh_) was defined as the minimum current required to elicit an action potential during a 2-ms current injection. The post-burst AHP was triggered using 25 brief (2 ms) somatic current injection (1 nA) at 50 Hz. Membrane properties including fast AHP (fAHP), medium AHP (mAHP), and slow AHP (sAHP) were assessed as previously described (Kaczorowski et al., 2007, 2011; Kaczorowski and Disterhoft, 2009; Neuner et al., 2015; Graves et al., 2016). Briefly, the mAHP was measured as the peak negative membrane potential relative to baseline and the sAHP was measured as the average negative membrane potential relative to baseline at 1–1.05 s after last brief current injection of protocol for triggering post-burst AHP. The fAHP was measured as the peak negative membrane potential following the action potential(s) in response to 2-ms current injection at threshold, relative to baseline. Repetitive firing was evoked by short (1-s) depolarizing current steps in 50-pA increments or by sustained (15 s) depolarizing current steps in 20-pA increments. The relationship between the firing frequency and injected current was plotted for each cell.

### Statistics

Data were analyzed using SPSS Statistics (IBM; RRID:SCR_002865) or R and visualized in R (RRID:SCR_001905). Behavioral data were analyzed using one-way ANOVA to identify differences between behavioral paradigms on freezing. Electrophysiological data were analyzed using univariate ANOVA (RMP, R_input_, I_thresh_, AHP) between behavioral paradigms and cell type unless otherwise indicated. To test for significant differences between groups, *post hoc* Bonferroni tests were applied and adjusted for multiple comparisons. Total spike data were non-normally distributed and, as such, underwent log transformation before analysis. Because behavioral and electrophysiological data were collected across two institutions, we (1) analyzed the results of behavioral training and testing and found no statistically significant effect of institution on any of the measures, and (2) included location of testing in the univariate ANOVA model and identified input resistance in BS as the only parameter that showed a significant interactive effect of recording location and behavioral treatment. Differences in intrinsic excitability between RS and BS cells in naïve animals were analyzed using independent samples *t* test. *Post hoc* power estimates were completed using G*Power software [Dusseldorf, Germany (Faul et al., 2007); RRID:SCR_013726] to confirm sufficient statistical power to perform analyses. A summary of statistical tests performed is shown in Table 1; superscript letters throughout the results section indicate corresponding statistic in this table.

**Table 1.** Detailed statistics summary

	Figure	Comparison	Data structure	Type of test	Statistic	Confidence, 95% CI
a	1*B*	Training freezing	Normal (except for FC-NS, ISD, ISD-NS)	One-way ANOVA	Df = 3, *F* = 57.015	*p* < 0.001
b	1*B*	Test freezing	Non-normal	One-way ANOVA	Df = 3, *F* = 61.116	*p* < 0.001
c	2*A*	RMP	Normal (except for FC-NS, ISD-NS)	One-way ANOVA	Df = 4; *F* = 1.737	*p* = 0.143
d	2*B*	R_input_	Non-normal	One-way ANOVA	df = 4, *F* = 2.099	*p* = 0.082
e	2*C*	fAHP	Normal	One-way ANOVA	df = 4, *F* = 2.581	*p* = 0.038
		Naïve-FC		*Post hoc* (Bonferroni)		*p* = 0.004; CI: –1.00 to –0.12
		Naïve-ISD		*Post hoc* (Bonferroni)		*p* = 0.001; CI: –1.09 to –0.18
		Naïve-ISD-NS		*Post hoc* (Bonferroni)		*p* = 0.003; CI: –1.03 to –0.14
f	2*D*	mAHP	Normal (except for FC, ISD)	One-way ANOVA	df = 4, *F* = 2.433	*p* = 0.049
		Naïve-FC		*Post hoc* (Bonferroni)		*p* < 0.000; CI: –3.41 to –1.07
		Naïve-ISD		*Post hoc* (Bonferroni)		*p* < 0.000; CI: –4.24 to –1.83
		Naïve-FC-NS		*Post hoc* (Bonferroni)		*p* = 0.015; CI: –2.81 to –0.17
g	2*E*	sAHP	Normal (except for ISD)	One-way ANOVA	df = 4, *F* = 2.26	*p* = 0.064
h	3*A*	I_thresh_	Normal (except for FC-NS, ISD)	One-way ANOVA	df = 4, *F* = 1.567	*p* = 0.18
i	3*B*	1-s current injection	Non-normal (log-transformed)	Repeated measures ANOVA	df = 4, *F* = 2.126	*p* = 0.079
j	3*B*	15-s current injection	Non-normal (log-transformed)	Repeated measures ANOVA	df = 4, *F* = 2.945	*p* = 0.021
		Naïve-FC		*Post hoc* (Bonferroni)		*p* = 0.30; CI: –0.75 to –0.02
		Naïve-ISD		*Post hoc* (Bonferroni)		*p* = 0.022; CI: –0.79 to –0.03
		Naïve-FC-NS		*Post hoc* (Bonferroni)		*p* = 0.20; CI: –0.88 to –0.04
		Naïve-ISD-NS		*Post hoc* (Bonferroni)		*p* = 0.035; CI: –0.75 to –0.02
k	4*A*	RMP	Normal (except for FC)	One-way ANOVA	df = 4, *F* = 1.166	*p* = 0.33
l	4*B*	R_input_	Normal (except for ISD, ISD-NS, Naïve)	One-way ANOVA	df = 4, *F* = 1.455	*p* = 0.22
m	4*C*	fAHP	Normal	One-way ANOVA	df = 4, *F* = 0.611	*p* = 0.66
n	4*D*	mAHP	Normal	One-way ANOVA	df = 4, *F* = 0.172	*p* = 0.952
o	4*E*	sAHP	Normal (except for ISD-NS)	One-way ANOVA	df = 4, *F* = 0.119	*p* = 0.119
p	5*A*	I_thresh_	Normal (except for ISD)	One-way ANOVA	df = 4, *F* = 0.36	*p* = 0.84
q	5*B*	1-s current injection	Non-normal (log-transformed)	Repeated measures ANOVA	df = 4, *F* = 1.214,	*p* = 0.311
r	5*B*	15-s current injection	Non-normal (log-transformed)	Repeated measures ANOVA	df = 4, *F* = 1.585	*p* = 0.186
s	6*B*	Naïve-FC cell-type proportions		Marasculio procedure (repeated χ^2^)	Test statistic value = 0.25	Critical value = 0.11
		Naïve-ISD cell-type proportions			Test statistic value = 0.21	Critical value = 0.11
		Naïve-FC-NS cell-type proportions			Test statistic value = 0.16	Critical value = 0.12
		Naïve-ISD-NS cell-type proportions			Test statistic value = 0.05	Critical value = 0.12

#### Exclusions

Cells with an RMP of –58 mV or higher were excluded from further recording. Cells were excluded for a lack of action potential firing (two cells), or after being identified as statistical outlier on all firing rate measures using a Grubb’s outlier test (one cell). Additionally, cells were excluded if their input resistance was indicative of interneurons rather than pyramidal neurons (24 cells), operationally defined as cells with an input resistance of higher than 3SD above the mean.

## Results

### Behavioral results

To study neuronal mechanisms specifically related to contextual fear memory formation, we performed contextual FC along with a variety of control paradigms designed to control for peripheral aspects including shock exposure and exposure to a novel context (Fig. 1*A*). As expected, animals subjected to the full training protocol during contextual FC (four foot shocks over 10 min; FC group) froze significantly more during training^a^ as well as 24 h after training^b^ when reintroduced to the training context, as compared both to animals from an ISD control group and two groups of no-shock controls (ISD no-shock, ISD-NS; and FC no shock, FC-NS (Fig. 1*B*); FC: 54.54 ± 4.49% freezing; ISD: 14.10 ± 3.83% freezing, *p* < 0.001; FC-NS: 1.33 ± 0.39% freezing, *p* < 0.000; ISD-NS ± 2.61 ± 1.64, *p* < 0.000; *n* = 11–16 animals per group). Animals in the ISD group were only exposed to a single 4-s-long foot shock, which began 1 s after they entered the chamber, and were removed within 30 s following shock. As a 1-s preshock period is not considered enough time for the animals to encode the training chamber context (Fanselow, 1986), ISD animals form a weak context-shock association compared to the FC animals. This is reflected by their significantly reduced freezing on testing, which is similar to mice that were exposed to the training chamber but never received foot shocks (FC-NS).

### Behavioral paradigm preferentially alters excitability in RS cells in the subiculum

We recorded from a total of 321 neurons across 54 mice [FC (*n* = 75 cells/13 animals), ISD (*n* = 66/12), FC-NS (*n* = 48/8), ISD-NS (*n* = 92/15), naïve (*n* = 43/6)]. To ensure consistency with previous studies, we first assessed differences in intrinsic membrane properties between RS and BS cells in naïve animals. RS and BS cells had similar intrinsic membrane and excitability properties as reported previously (Staff et al., 2000; Graves et al., 2012), although we also found that BS neurons exhibited significantly enhanced fAHP (RS: –1.17 ± 0.16 mV, BS: –2.88 ± 0.36 mV, *p* < 0.001 by independent samples *t* test). RS and BS had similar R_input_, I_thresh_, mAHP and sAHP, and RMP (Table 2).

**Table 2.** Intrinsic properties of RS versus BS subicular neurons from naïve animals

	RS	BS	*p* Value
RMP (mV)	–63.6 ± 0.71	–65.4 ± 0.67	0.07
R_input_ (MOhm)	118.0 ± 12.8	94.5 ± 5.6	0.10
I_thresh_ (pA)	802.5 ± 47.5	689.5 ± 34.5	0.06
fAHP (mV)	–1.17 ± 0.16	–2.89 ± 0.36	<0.001***
mAHP (mV)	–4.81 ± 0.55	–4.92 ± 0.68	0.90
sAHP (mV)	–0.61 ± 0.07	–0.47 ± 0.06	0.14

**Table 3.** Degree of contextual learning is significantly associated with greater excitability in RS but not BS cells

	RS	BS
RMP	R = –0.30 *p* = 0.026	R = –0.45 *p* = 0.11
Rinput	R = 0.009 *p* = 0.945	R = 0.25 *p* = 0.41
Ithresh	R = –0.43 *p* = 0.006*	R = –0.06 *p* = 0.83
fAHP	R = 0.03 *p* = 0.64	R = 0.12 *p* = 0.30
mAHP	R = 0.37, *p* = 0.003*	R = 08.45 *p* = 0.10
sAHP	R = 0.08 *p* = 0.52	R = 0.07 *p* = 0.81
Firing (1 s)	R = 0.25, *p* = 0.048	R = 0.38 *p* = 0.18
Firing (15 s)	R = 0.24 *p* = 0.073	R = 0.23 *p* = 0.41

A Bonferroni correction for multiple comparisons was applied to the significance level of correlation coefficients; *p < 0.0064.

**Figure 2.**
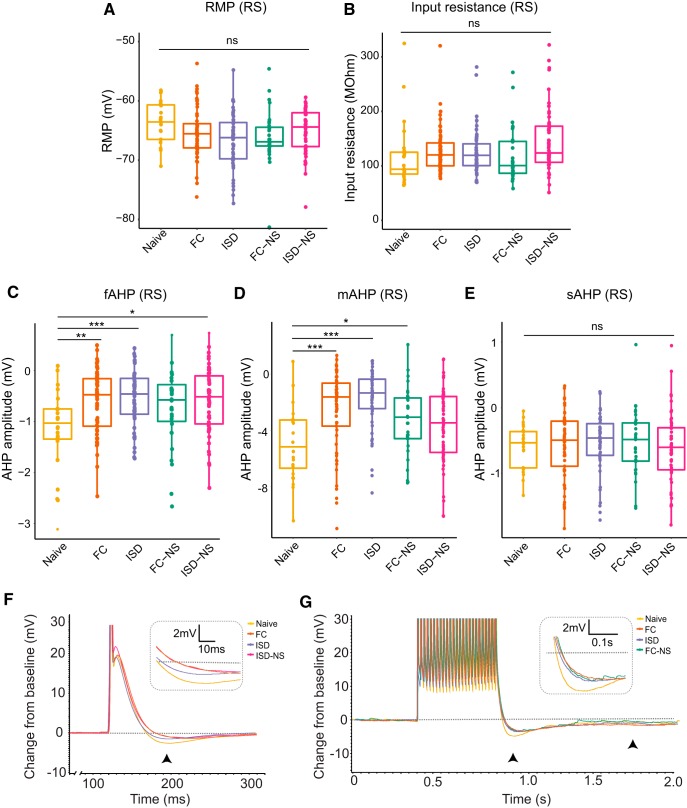
Exposure to behavioral training enhances excitability of neurons in RS cells as measured by post-spike and post-burst AHP. In RS cells, no significant main effect of behavior on (***A***) RMP or (***B***) R_input_ was observed. ***C***, However, the fAHP following an action potential elicited by a 2-ms current injection was significantly modulated by behavioral training exposure. Naïve (untrained) animals exhibited the most negative fAHP, indicating reduced excitability. ***D***, Behavioral training also reduced the post-burst (25-spike) mAHP, with naïve animals again exhibiting the most negative mAHP (least excitable). ***E***, The post-burst (25-spike) sAHP was not significantly modulated by behavioral paradigm. ***F***, Representative traces from naive, FC, and ISD animals indicate less negative fAHP (black arrowhead; inset) in FC and ISD groups. Groups with significant differences from naïve are shown. ***G***, Representative traces from naive, FC, and ISD animals indicate less negative mAHP (left arrowhead; inset) and unaltered sAHP (right arrowhead) in FC and ISD groups. Groups with significant differences from naïve are shown; *p < 0.05; **p < 0.01, ***p < 0.001.

To determine whether RS and BS subicular pyramidal neurons undergo experience-dependent plasticity of intrinsic membrane properties, we next compared RMP, R_input_, I_thresh_, and AHP (fast, medium, and slow) within each cell type across behavioral paradigms. In RS cells, we found no significant main effect of behavior on RMP (df = 4, *F* = 1.737, *p* = 0.143^c^; Fig. 2*A*), R_input_ (df = 4, *F* = 2.099, *p* = 0.082^d^; Fig. 2*B*). However, RS cells displayed significant experience-dependent changes in other intrinsic measures of excitability following behavioral paradigms. Specifically, in RS cells, we observed a significant main effect of behavioral group on both the fAHP (df = 4, *F* = 2.581, *p* = 0.038^e^; Fig. 2*C*,*F*) and mAHP (df = 4, *F* = 2.433, *p* = 0.049^f^; Fig. 2*D*,*G*), although there was no significant effect of behavioral group on sAHP (df = 4, *F* = 2.26, *p* = 0.064^g^; Fig. 2*E*,*G*). Naive animals exhibited the lowest excitability across these parameters, demonstrating that exposure to new environmental conditions or learning experiences is sufficient to remodel subicular RS neurons.

**Figure 3.**
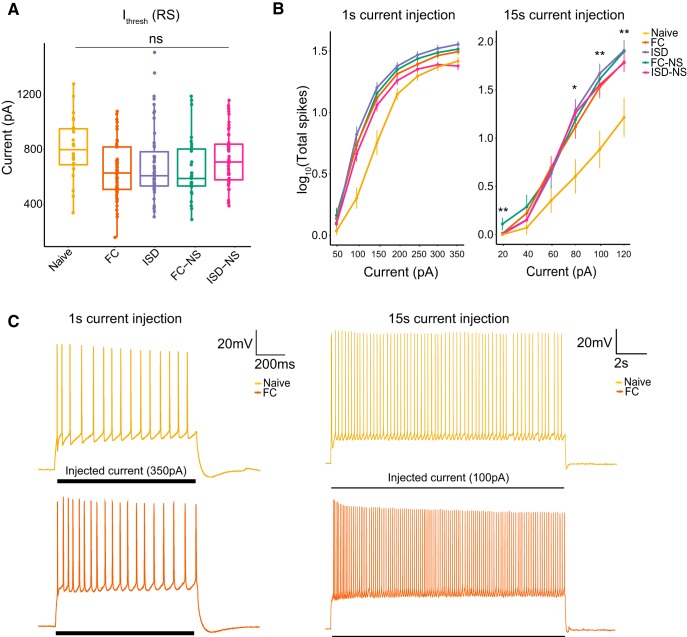
Behavioral training results in higher firing rate in RS cells during a 15-s current injection. ***A***, There was no main effect of behavior on I_thresh_ in RS cells. ***B***, There was a significant main effect of behavior on firing rate in response to a (right) 15-s, but not (left) 1-s, current injection of increasing amplitude, with naïve animals and displaying the fewest total spikes during the current injection paradigms. ***C***, Representative current traces from naïve and FC animals demonstrate firing rate during a 350-pA current injection (1 s) or 100-pA current injection (15 s); *p < 0.05; **p < 0.01; naïve, *n* = 24 cells; FC, *n* = 61; ISD, *n* = 50; FC-NS, *n* = 31; ISD-NS, *n* = 55.

In RS cells, we did not find a significant effect of behavioral exposure on I_thresh_ (df = 4, *F* = 1.567, *p* = 0.18^h^; Fig. 3*A*), nor on total spikes during a 1 s (df = 4, *F* = 2.126, *p* = 0.079^i^; Fig. 3*B*, left) current injection. In contrast, we observed a significant effect of training exposure on total spikes fired during a 15-s current injection in RS cells (df = 4, *F* = 2.945, *p* = 0.021^j^; Fig. 3*B*, right) and *post hoc* pairwise tests revealed a significant difference between naïve animals and all other groups (FC: *p* = 0.03; ISD: *p* = 0.02, FC-NS: *p* = 0.02; ISD-NS: *p* = 0.04). Representative voltage responses demonstrate a similar number of spikes in naïve and FC-trained animals during a 1 s, 350-pA current injection (Fig. 3*C*, left), and an increased number of spikes during a 15-s, 100-pA current injection in FC-trained animals compared to naïve animals (Fig. 3*C*, right). In general, we observed a trend toward reduced excitability on each of these measures in naïve animals compared to animals that underwent exposure to the conditioning chambers, with or without shock.

**Figure 4.**
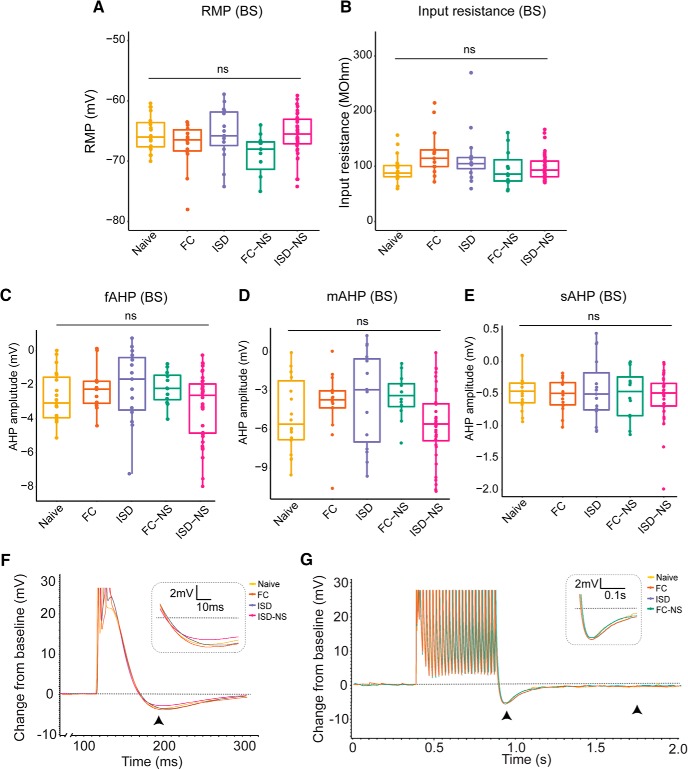
Exposure to behavioral training does not significantly enhance excitability of BS cells. ***A***, ***B***, We observed no significant main effect of behavioral exposure on the RMP or R_input_ of BS cells. ***C***, The fAHP following an action potential elicited by a 2-ms current injection was not significantly modulated by behavioral training exposure in BS cells. ***D***, ***E***, Similarly, no significant main effect of behavior was observed on the post-burst (25-spike) mAHP or sAHP. ***F***, Representative traces from naive, FC, and ISD animals indicate similar fAHP (black arrowhead; inset) in naïve, FC, and ISD groups. Groups with significant differences from naïve in RS cells are included. ***G***, Representative traces from naive, FC, and ISD animals also indicate similar mAHP (left arrowhead; inset) and sAHP (right arrowhead) in naïve, FC, and ISD groups. Groups with significant differences from naïve in RS cells are included; ns = not significant.

**Figure 5.**
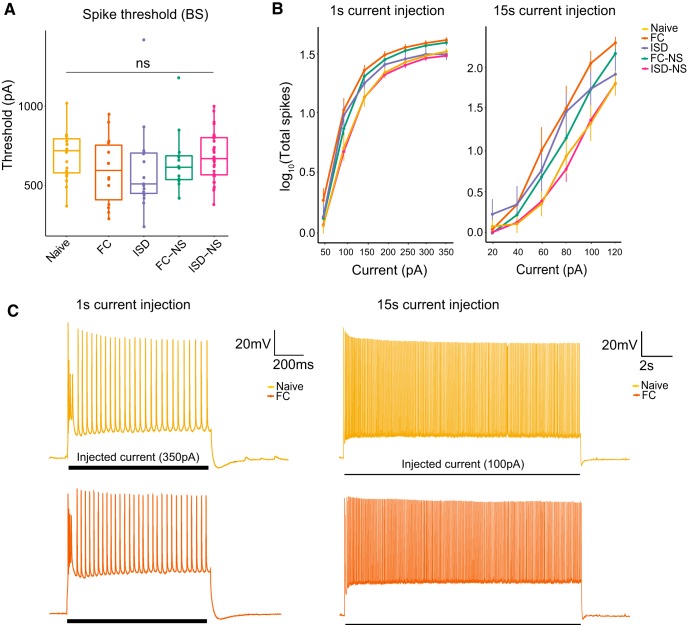
Behavioral training does not significantly modulate propensity to fire in BS cells. ***A***, We observed no significant main effect of behavior on (***A***) threshold to elicit an action potential or (***B***) firing rate in response to a 1- or 15-s current injection of increasing amplitude. ***C***, Representative current traces from naïve and FC animals demonstrate firing rate during a 350-pA current injection (1 s) or 100-pA current injection (15 s); naïve, *n* = 19 cells; FC, *n* = 14; ISD, *n* = 15; FC-NS, *n* = 12; ISD-NS, *n* = 36.

In BS cells, we observed no significant main effects of behavior on any measure of excitability (RMP: df = 4, *F* = 1.166, *p* = 0.33^k^; R_input_: df = 4, *F* = 1.455, *p* = 0.22^l^; fAHP: df = 4, *F* = 0.611, *p* = 0.66^m^; mAHP: df = 4, *F* = 0.172, *p* = 0.952^n^; *p* = 0.66; sAHP: df = 4, *F* = 0.119, *p* = 0.975^°^; I_thresh_: df = 4, *F* = 0.36, *p* = 0.84^p^; spikes during a 1-s current injection: df = 4, *F* = 1.214, *p* = 0.311^q^; spikes during a 15-s current injection: df = 4, *F* = 1.585, *p* = 0.186^r^; Figs. 4, 5). This suggests RS cells are relatively more responsive to environmental-induced changes, which may indicate that RS cell plasticity plays a particularly important role in regulating salient contextual information flow from the hippocampus to cortical regions.

### Degree of contextual learning predicts greater excitability in RS, but not BS, cells from FC animals

**Figure 6.**
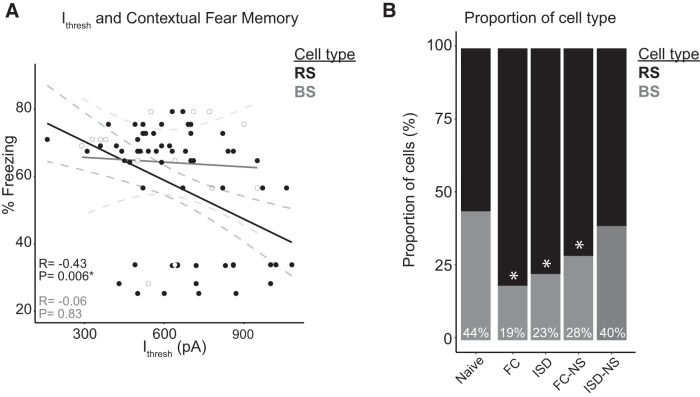
RS cell excitability is associated with greater learning of contextual fear memory, and contextual FC results in a reduction of BS cells. ***A***, There is a significant negative correlation between the threshold to elicit a spike in RS, but not BS, cells. ***B***, The proportion of BS cells is decreased significantly following FC, ISD, and FC-NS exposures, with the mildest training exposure (ISD-NS) displaying a similar proportion of BS cells to naïve animals; *p < 0.05.

Contextual fear memory acquisition and retrieval are dependent on several memory-relevant brain structures, including the hippocampus, subiculum, and entorhinal cortex, as well as fear-relevant structures such as the amygdala and mammillary bodies. The subiculum regulates information transmission to each of these circuits (Roy et al., 2017). To evaluate whether greater contextual learning (i.e., more freezing during the context test following training) correlated with greater intrinsic excitability in subicular neurons in the FC group, we performed Pearson's correlation tests between all membrane properties and excitability measures of RS and BS neurons and the percentage freezing during testing (Table 3; Fig. 6*A*). In BS cells, degree of freezing did not significantly correlate with any membrane or excitability properties. In RS cells, freezing was significantly negatively correlated with I_thresh_ in FC animals (*r* = –0.43, *p* = 0.006*), and significantly positively correlated with mAHP (*r* = 0.45, *p* = 0.003*; *α Bonferroni-adjusted to 0.0064 for multiple comparisons). This indicates that the degree of learning-induced subicular plasticity predicted contextual fear memory performance (i.e., better learning was associated with greater subicular neuron plasticity and excitability, as indicated by decreased input required to fire an action potential and quicker rebound after action potential firing as indicated by decreased mAHP).

### Ratio of BS to RS cells in the subiculum varies with novel contextual experience

Consistent with previous reports (Staff et al., 2000; Graves et al., 2012, 2016), nearly half of the subicular neurons recorded in brain slices from naïve mice exhibited BS firing. Surprisingly, however, when we assessed the ratio of BS to RS neurons in each behavioral paradigm (Fig. 6*B*), we found that behavioral training significantly altered the proportion of BS to RS cells. Mice in the FC group that were trained and re-exposed to the conditioning context exhibited the lowest proportion of BS cells (18.7%), followed by the ISD group (23.1%) and the FC-NS group (27.9%). By a Marascuilo procedure^s^ (repeated χ^2^), this proportion was significantly different from the naïve group following each of these exposures. Among animals that underwent behavioral testing, the proportion of BS cells was highest in animals in the ISD-NS group (39.6%); this proportion was comparable that seen in naive animals (44.2%). Together, these results suggest that not only does successful learning and memory on a contextual FC task robustly alter the burst firing properties of subicular pyramidal neurons, but that milder conditioning and novel environment exposure induces similar changes, although to a lesser extent (Fig. 6).

## Discussion

Here, we provide the first direct evidence that subicular neurons undergo cell-type-specific learning- and experience-related plasticity in intrinsic properties and that this plasticity is driven both by specific enhanced excitability of RS cells and an apparent conversion of BS to RS activity.

### Cell-type specificity in intrinsic properties plasticity following novel context exposure and FC

We observed general enhancement of excitability in subicular neurons following exposure to a novel context and a FC paradigm, although this differed by cell type. Specifically, we found that RS cells displayed greater plasticity in intrinsic excitability compared to BS cells, and that a greater degree of excitability in RS cells, but not BS cells, was significantly associated with a better contextual fear memory performance. Our findings are consistent with previous work showing that learning and novel environments modulate intrinsic excitability, and that the degree of learning is predictive of the magnitude of neuronal excitability changes in CA1 (Kaczorowski and Disterhoft, 2009; Matthews et al., 2009; Oh et al., 2010; Kaczorowski et al., 2011; Song et al., 2012). We demonstrate here for the first time that subicular neurons also undergo experience-dependent intrinsic plasticity and that these changes are cell-type specific.

Previous literature has shown cell-type differences in synaptic plasticity in RS and BS cells, particularly in terms of mechanisms of LTP and LTD in CA1-subicular synapses (Fidzinski et al., 2008; Moore et al., 2009; Shor et al., 2009; Graves et al., 2012, 2016). As changes in intrinsic excitability can modulate efficacy of synaptic plasticity, it is possible that our observed intrinsic plasticity is in part responsible for differences previously observed in synaptic plasticity in projections to RS and BS cells. Discrete populations of subicular cells differ in expression of various genes that likely play role in regulating intrinsic excitability, such as voltage-gated potassium and calcium channels (Cembrowski et al., 2018), which may represent potential cell-type-specific targets of neuronal excitability modulation. Both the fAHP and mAHP are mediated by potassium and calcium currents, as well as internal calcium regulation (Storm, 1987, 1989, 1990; Brown et al., 1990). The fAHP is generated following calcium influx during the action potential, which activates large-conductance potassium channels and calcium and voltage-dependent BK currents that repolarize the cell. Similarly, the mAHP is generated following calcium influx during one or several action potentials followed by potassium and other cation efflux via SK channels, M-channels and h-channels (Gu et al., 2005; Kaczorowski, 2011; Chen et al., 2014), although the precise mechanisms have been debated and likely are not yet fully understood. The currents underlying all three AHPs are plastic and may be altered by experience and learning (Saar et al., 1998; Zhang and Linden, 2003; Matthews et al., 2008, 2009; Chen et al., 2014; Whitaker et al., 2017), although the differences in regulation of AHPs in RS versus BS cells have not been investigated. Our data suggest that regulation learning-related plasticity in the fAHP and mAHP may be cell-type dependent. Targeting intrinsic plasticity in a cell-type-specific manner in the subiculum (i.e., specifically enhancing excitability of RS cells to improve learning) may thus be an effective way to modulate cognitive function in learning- and memory-related disorders, such as dementia.

### Increased proportion of RS cells with novel context exposures

Unexpectedly, we found that behavioral training resulted in a reduction in the proportion of BS cells and overrepresentation of RS cells within the subiculum. This was in a training-intensity dependent manner, where cells from animals who underwent the most intense training followed by contextual recall (FC) had a 57% reduction in the proportion of BS cells compared to naïve animals. ISD and FC-NS also resulted in a reduction in BS:RS, albeit a more modest reduction (47% and 36% reduction from naïve baseline, respectively). ISD-NS animals had the mildest training exposure, and showed a similar proportion of BS:RS cells to naïve animals. This suggests that fear learning and/or encoding of new contexts is facilitated by a conversion of subicular BS to RS cells, and that acute exposure to novel contexts alone, in addition to undergoing a learning paradigm, is sufficient to begin to remodel subicular neuron intrinsic properties. Because most BS cells are in the distal subiculum, we expect that this is specifically reflective of plasticity in distal subicular cells to modulate firing behavior based on context. Recent publications have demonstrated that the distal subiculum is required for spatial working memory and behavioral adaptation to context, and that the distal subiculum is specifically critical to retrieval (but not encoding) of contextual fear memories (Roy et al., 2017; Cembrowski et al., 2018). Our observation that BS cells (which are enriched in the distal subiculum) appear to convert to RS cells to the greatest degree in the FC group is consistent with the distal subiculum being preferentially involved in hippocampal-dependent spatial and contextual memory retrieval.

It is important to note that behavioral testing was randomized and electrophysiological recordings were performed by experimenters blinded to behavioral condition. Further, the sampling procedure of cells at random along the entire axis of the subiculum conforms to multiple studies by our group and others, which consistently identifies ∼50% BS cells in the subiculum in naïve animals (Staff et al., 2000; Jarsky et al., 2008; Graves et al., 2016). We also identified approximately half of cells as BS in naïve animals (44%; Fig. 6), bolstering the reproducibility of these findings. Therefore, it is unlikely our findings relating specific experience-dependent changes in proportion of BS cells were confounded by oversampling of the distal subiculum.

### Implications and mechanisms for a change in bursting activity in the subiculum

The observed change in the ratio of RS:BS cells within the subiculum following behavioral paradigms supports the notion that RS and BS activity convey specialized information related to learning, environmental context, and behavior. BS allows for a higher probability of signal transmission in the postsynaptic cell, and is therefore valuable in conveying information in a robust manner. However, our observation that RS cells are subject to greater plasticity following learning and novel context exposure may indicate that RS cells allow for finer-tuning of information flow that may be particularly important during active learning and retrieval, when signal transmission flexibility is crucial. Additionally, intrinsic excitability is relatively more plastic and associated with learning in RS cells, which may better poise RS cells to respond and encode information about novel experiences. Alternatively, synaptic strengthening that occurs during learning and recall may allow for a similar strength response from RS that previously required BS input, and thus RS activity may be sufficient to elicit the same effect on downstream targets after learning. Finally, it is also possible BS cells that were recruited during learning better maintain their propensity to burst, while BS cells not participating in context encoding may experience a reduction in burst propensity, thereby enhancing the signal of the remaining/participatory BS cells over those cells that did not participate.

Previous studies have suggested that BS and RS cells are distinct populations in discrete pathways that are not interconvertible in slice preparations (Graves et al., 2012; Kim and Spruston, 2012). Additionally, transcriptional profiles are distinct between proximal and distal subicular neurons, and this distinction presumably extends to RS and BS neurons (Cembrowski et al., 2018). Recent work has suggested, however, that there is collateralization of projections that may indicate a greater degree of diversity and potential for flexibility in projection targets from RS and BS cells than previously thought (Cembrowski et al., 2018). The ability to burst also seems to be an intrinsic property of all subicular neurons primarily mediated by calcium currents via R-type and T-type calcium channels that open during depolarization to allow calcium influx (Jung et al., 2001; Su et al., 2002; Yue and Yaari, 2004), and bursting is sensitive to changes in potassium currents (Jensen et al., 1994; Staff et al., 2000; Hu et al., 2017). This suggests that a remodeling of ion channel populations and activity *in vivo* during learning or novel context exposure may be a candidate mechanism for changes in bursting activity in the subiculum. Age and status epilepticus have also been shown to change proportion of BS versus RS in the subiculum (Wellmer et al., 2002; Knopp et al., 2005; Chung et al., 2015), and other evidence suggests that such activity-dependent changes in bursting activity are mediated by T-type calcium channels (Su et al., 2002).

Previous work has demonstrated cell-type-specific modulation of both intrinsic and synaptic plasticity in the subiculum (Moore et al., 2009; Graves et al., 2012, 2016). Specifically, bath application of BDNF enhances intrinsic excitability of BS neurons while reducing excitability of RS neurons (Graves et al., 2016). Similarly, glutamatergic and cholinergic inputs bidirectionally modulate excitability depending on cell type and input composition, with cholinergic and glutamatergic inputs acting synergistically to enhance bursting in BS neurons but glutamatergic signaling via mGluR5 acting to suppress bursting in BS cells in the absence of concurrent cholinergic inputs (Moore et al., 2009; Graves et al., 2012). In addition to their role in mediating burst propensity, mGluR5 activation also results in reduction of the AHP and enhances intrinsic excitability of cortical RS neurons (Sourdet et al., 2003). These studies were completed exclusively in behaviorally naive rodents, however, and it is unclear how learning and memory formation *in vivo* may involve these processes. Based on these previous models, it is possible that circuitry activation during learning and memory recall preferentially induces signaling via mGluR5 receptors to suppress bursting in BS cells and facilitate excitability of RS cells/RS activity. mGluR5 activation is critical to learning and memory, with genetic knockout and pharmacological antagonism of mGluR5 impairing performance on hippocampal-dependent spatial learning and memory tasks (Xu et al., 2009; Simonyi et al., 2010). Conversely, enhancement of mGluR5 signaling via a specific positive allosteric modulator enhances performance on contextual FC specifically (Sethna and Wang, 2014). Together with our data, this suggests that in addition to ion channel composition, possible mGluR5-mediated enhancement of RS activity in the subiculum is important for regulating hippocampal output in learning and memory. Future studies may investigate changes in ion channel density and mGluR5 signaling in the subiculum following learning, to account for the observed flexibility in RS versus BS signaling following learning *in vivo*.

Because we observed a concurrent enhancement of intrinsic excitability and reduction in burst firing following FC and novel context exposure, it is likely that measures of intrinsic excitability and propensity to burst are two forms of plasticity that may be regulated separately. Indeed, burst propensity and other measures of intrinsic excitability are distinctly governed, which may allow for finer-tuning of neuronal remodeling in the subiculum following learning and novel context exposure.

### Plasticity within the cubiculum regulates spatial information output from the hippocampus

In conclusion, we demonstrate here for the first time that subicular neurons undergo cell-type-specific plasticity in intrinsic excitability following learning and novel context exposure. Interestingly, an unexpected additional form of plasticity appears to be a change in ratio of BS:RS cells following behavioral training, and memory consolidation and recall. The ability of the subiculum to regulate hippocampal output via an increase or decrease in BS:RS activity *in vivo* following learning likely represents a novel form of plasticity not previously observed in slice preparations that provides further insight to our understanding of the precise role of the subiculum in learning and memory.

## Synthesis

Reviewing Editor: Christian Hansel, University of Chicago Decisions are customarily a result of the Reviewing Editor and the peer reviewers coming together and discussing their recommendations until a consensus is reached. When revisions are invited, a fact-based synthesis statement explaining their decision and outlining what is needed to prepare a revision will be listed below. Note: If this manuscript was transferred from JNeurosci and a decision was made to accept the manuscript without peer review, a brief statement to this effect will instead be what is listed below.

The authors have done a great job in revising their manuscript for eNeuro in response to the previous reviewer comments at JN.

For this reason, no further review is required.
